# Integrating optical coherence tomography and bioluminescence with predictive modeling for quantitative assessment of methicillin-resistant *S. aureus* biofilms

**DOI:** 10.1117/1.JBO.30.S3.S34111

**Published:** 2025-09-23

**Authors:** Valentin V. Demidov, Olivia P. Jackson, Natalia Demidova, Jason R. Gunn, I. Leah Gitajn, Jonathan Thomas Elliott

**Affiliations:** a Dartmouth-Hitchcock Medical Center, Department of Orthopaedics, Lebanon, New Hampshire, United States; b Dartmouth College, Geisel School of Medicine, Hanover, New Hampshire, United States; c Dartmouth College, Thayer School of Engineering, Hanover, New Hampshire, United States

**Keywords:** bioluminescence imaging, biofilm, methicillin-resistant *Staphylococcus aureus*, dual-modality imaging, optical coherence tomography

## Abstract

**Significance:**

Methicillin-resistant *Staphylococcus aureus* (MRSA) biofilm infections present a critical challenge in orthopedic trauma surgery and are notoriously resistant to systemic antibiotic therapy. Noninvasive, quantitative imaging methods are urgently needed to assess biofilm burden and therapeutic efficacy, especially for emerging photodynamic therapy (PDT) strategies.

**Aim:**

We aim to establish a quantitative framework using a combined bioluminescence and optical coherence tomography (OCT) imaging approach to correlate bioluminescent signal with viable MRSA burden in both planktonic and biofilm states and to determine how biofilm density and structure influence this relationship.

**Approach:**

Bioluminescent MRSA (SAP231-luxCDABE) was cultured in planktonic and biofilm forms using *in vitro* growth models in 24-well plates and custom macrofluidic devices, respectively. Bacteria bioluminescence intensity (BLI), counted colony-forming units (CFU), and OCT-based biofilm thickness measurements were collected to construct linear regression models to evaluate how well BLI alone, or combined with biofilm density (CFU/volume), predicts bacterial counts across culture conditions.

**Results:**

Bioluminescence strongly correlated with CFU in planktonic cultures ($R^{2}=0.98$). In biofilms, BLI per CFU decreased with density, indicating metabolic downregulation, and BLI alone was less reliable ($R^{2}=0.59$). Incorporating biofilm density (CFU/volume) improved prediction ($R^{2}=0.84$). A joint model for both states showed excellent fit ($R^{2}=0.985$), but the biofilm versus planktonic group remained a significant factor (p=0.002), revealing systematic differences. This highlights the need for a mixed-model approach that segments subvolumes by morphological features to improve accurate, generalizable CFU estimation across both growth states.

**Conclusions:**

Bioluminescence alone underestimates bacterial burden in dense, metabolically suppressed MRSA biofilms. The combination of BLI with OCT-derived structural metrics enables accurate, nondestructive quantification of viable bacterial load. This approach provides a robust toolset for preclinical evaluation of antimicrobial therapies, particularly for optimizing PDT dosimetry and assessing biofilm response in translational infection models.

## 1 Introduction

Orthopedic trauma surgeries frequently involve hardware implantation, especially in cases of complex fractures and limb salvage procedures. Postoperative infection remains a serious complication, with reported rates ranging from 5% to 30%, depending on injury severity and treatment conditions.^1^ A substantial proportion of these infections are caused by methicillin-resistant *Staphylococcus aureus* (MRSA), which readily forms biofilms on implanted surfaces and within peri-implant tissues.^2^ These biofilms shield bacteria from host immune defenses and systemic antibiotics, enabling chronic infection and recurrent dissemination of planktonic bacteria even after aggressive debridement and irrigation procedures with antimicrobial solutions.^3^

As antibiotic resistance continues to limit treatment options,^4^ alternative therapeutic strategies such as antimicrobial photodynamic therapy (aPDT) are being actively explored. aPDT utilizes a photosensitizer and visible light to generate reactive oxygen species that can kill bacteria and disrupt the extracellular biofilm matrix.^5^^,^^6^ Due to its localized action with minimal collateral damage to host tissues^7^ and low risk of resistance development, aPDT is a compelling candidate for managing biofilm-associated infections in orthopedics. However, the translational success of aPDT relies on rigorous preclinical testing and the ability to noninvasively quantify bacterial burden and treatment response.^8^

Conventional biofilm assays such as colony-forming unit (CFU) assays or crystal violet staining are invasive, destructive, and impractical for real-time or longitudinal monitoring of biofilms.^9^ By contrast, optical techniques offer noncontact, label-free, or genetically encoded ways to assess bacterial viability and biofilm structure. Bioluminescence imaging (BLI) using genetically engineered MRSA strains, such as the SAP231-luxCDABE strain, allows real-time visualization of metabolically active bacteria.^10^^,^^11^ The SAP231 strain, derived from the clinical isolate NRS384, harbors the luxCDABE operon, enabling self-sustained light emission from metabolically active cells.^12^ This strain has been widely adopted in preclinical orthopedic infection models due to its robust bioluminescence and biofilm-forming capacity. However, the expression of the bioluminescence gene luxCDABE is strongly influenced by bacterial metabolic state, which is often suppressed in mature or nutrient-deprived biofilms, potentially leading to underestimation of viable bacterial load.^13^^,^^14^ As a result, bioluminescence intensity alone may underestimate viable bacterial burden in mature or quiescent biofilms.

To overcome this limitation, we integrated optical coherence tomography (OCT) with BLI to provide a complementary structural assessment of biofilm architecture. OCT offers high-resolution, depth-resolved imaging of biofilm thickness and spatial heterogeneity,^15^ enabling estimates of biofilm volume and density—factors that critically influence the interpretation of BLI signals. In this *in vitro* study, we developed a dual-modality *in vivo* imaging system (IVIS)-OCT imaging platform to enhance the accuracy of MRSA quantification in both planktonic and biofilm forms. By integrating structural biofilm measurements from OCT with BLI from SAP231, we constructed predictive models that improve estimation of viable bacterial burden compared with bioluminescence alone. This optical framework directly supports the advancement of biophotonics-based antimicrobial strategies such as photodynamic therapy and represents a step toward more precise, real-time monitoring of infection dynamics in preclinical orthopedic models.

## 2 Methods

### 2.1 Planktonic MRSA Culture and Imaging

A freezer stock of MRSA strain SAP231 with bioluminescent protein LuxCDABE12 was stored in 80% glycerol in a centrifuge tube at −80°C. The bacteria were pelleted by centrifugation and resuspended in phosphate-buffered saline (PBS). 100  μL of the sample was removed and plated on a tryptic soy agar (TSA) plate with 10  μg/mL of chloramphenicol (Sigma, St. Louis, Missouri, United States) to be cultured overnight at 37°C. The plate was imaged using the Perkin Elmer *in vivo* imaging system (IVIS Spectrum, PerkinElmer, Shelton, Connecticut, United States) to verify bioluminescence; then, a single colony was selected with an inoculating loop and incubated in 10 mL tryptic soy broth (TSB, Thermo Fisher Scientific Remel Products, Lenexa, Kansas, United States) at 37°C 200 rpm overnight. The inoculate was prepared by making a 1:100 dilution of the overnight culture in TSB with 10  μg/mL chloramphenicol and allowed to culture for 3 h at 37°C, 200 rpm. After culturing for 3 h, 1 mL of the sample was measured using a spectrophotometer (ATO Inc., Diamond Bar, California, United States) to obtain an (OD)600 reading to approximate the concentration of the sample, in CFUs per 1 mL, using an assumption that a 1 mL sample of MRSA with an (OD)600 = 1.5 contains $10^{9}$ CFU.^12^

**Fig. 1:**
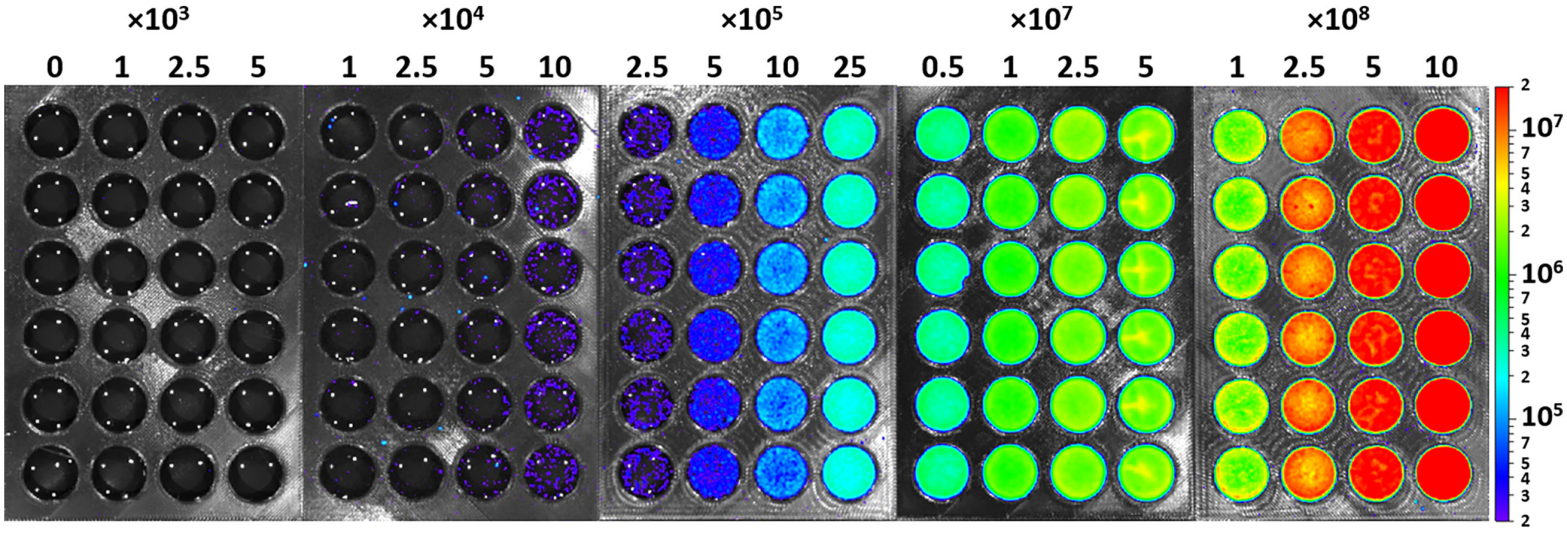
Planktonic MRSA bioluminescence in 500  μL TSB in 24-well plates, measured 2 min after pipetting into the wells. CFU counts per well are indicated at the top of each corresponding column (e.g., 0 CFU, $1\times 10^{3}\;\mathrm{CFU}$, and $2.5\times 10^{3}\;\mathrm{CFU}$). Bioluminescence units are in $\text{photon}/\sec/{\mathrm{cm}}^{2}/\text{steradian}$ (logarithmic scale).

Serial dilutions were performed on the completed MRSA culture using TSB. Twenty groups of 0.5 mL aliquots of a known number of CFU in TSB (n=6 per group, including controls with no bacteria) were pipetted into each row of the wells of five 24-well plates. The number of CFUs in each group ranged from $10^{3}$ to $10^{9}$ by orders of magnitude, increasing by a factor of 2.5, 5, or 10 each time, and were verified using OD(600) measurements. Each well plate was imaged using IVIS to measure the average radiance in $p/\sec/{\mathrm{cm}}^{2}/\mathrm{sr}$ units within a designated region of interest (ROI). Images were taken on level C of the IVIS with a stage height of 1.1 cm, with a lateral resolution of 385  μm. The exposure time was set to 60 s. Using Living Image 4.7.3 software (PerkinElmer Inc., Waltham, Massachusetts, United States), bioluminescence imaging data were presented on a color scale overlaid on a grayscale photograph, and circular ROIs of the same size ($2\;{\mathrm{cm}}^{2}$) and location were centered over each well to include the entire sample. Average bioluminescence intensity of the known number of planktonic CFUs was calculated as a mean value of the radiance of ROIs across all 6 wells (Fig. 1).

### 2.2 MRSA Biofilm Growth and Imaging

**Fig. 2:**
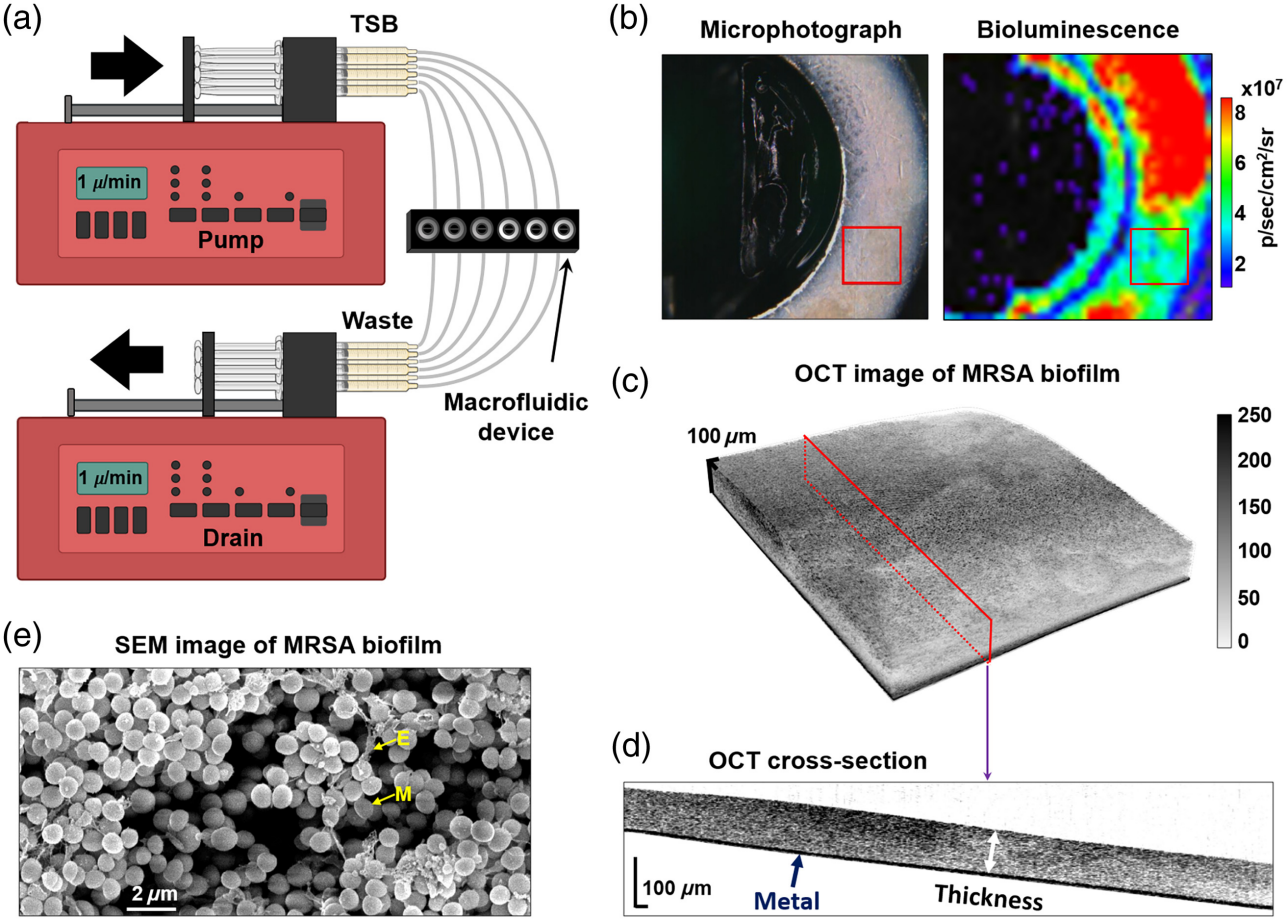
(a) Schematic of a macrofluidic model of MRSA biofilm growth. Black arrows indicate the direction of flow: the top pump pushes tryptic soy broth (TSB) through the microtubes to the wells of the macrofluidic device (MD), whereas the bottom pump simultaneously pulls the waste. (b) White light and corresponding bioluminescent image of a biofilm grown on a metal orthopaedic washer. Red rectangles indicate the area where the OCT image shown in panel (c) was acquired; (c) OCT 3D-rendered image of a $1.5\times 1.5\;{\mathrm{mm}}^{2}$ region of a biofilm; (d) cross sectional OCT image from the location labeled with red in panel (c); biofilm thickness was measured from the biofilm surface down to the metal surface as indicated with a white double arrow; (e) SEM image of MRSA biofilm. M, MRSA; E, extracellular polymeric substance.

A well-established procedure^16^ was implemented for MRSA biofilm growth on metal surfaces in macrofluidic devices to correlate bioluminescence with the amount of biofilm-state bacteria. Briefly: metal washers (titanium and stainless-steel, Stryker, Kalamazoo, Michigan, United States) were soaked in a 0.5% bleach solution, scrubbed with a soft bristle brush, and then dried and wrapped in sterilization paper to be autoclaved in a gravity displacement sterilizer for 30 min at 121°C before use. All six washers were then transferred to the 6-well custom-designed stainless steel macrofluidic device for growth under dynamic conditions with the flat, unetched face of the washer facing upward. Each macrofluidic device was fed and drained by two programmable syringe pumps (Braintree Scientific, Inc., Braintree, Massachusetts, United States) at a rate of 1  μL/min through #30 Ga microtubes (Avantor Fluid Handling, Devens, Massachusetts, United States), as shown schematically in Fig. 2(a). The 3 mL BD luer-lok syringes (Becton, Dickinson and Co., Franklin Lakes, New Jersey, United States) were filled with sterile TSB mixed with 5% FBS and 10  μg/mL chloramphenicol. For inoculation, the overnight MRSA culture was diluted with TSB to obtain a concentration of $10^{8}$ CFUs per 200  μL of culture and added to the 1.3 mL of the broth mixture in each well. After inoculation, the macrofluidic device was kept for 24 to 72 h (to achieve various growth and maturity stages) in a biosafety cabinet on a heating pad, set to maintain 34°C to 38°C device temperature. This range is within the optimal growth conditions for the bacterial species used and is not expected to introduce variability in growth or biofilm development.

Before imaging, a thin needle was used to gently penetrate the fluid surface at the edges of each well to detach any biofilm adhering to the walls. Washers were then removed one at a time, with care taken to minimize disruption to the biofilm. Each washer was immediately placed into a separate well plate and imaged individually to avoid desiccation and potential changes in biofilm density. During this process, fluid flow continued uninterrupted to the remaining washers within the macrofluidic device. For each washer, bioluminescence imaging was performed first, followed by OCT imaging of the same washer. This procedure was repeated consistently for all washers across all experiments to ensure reproducibility and minimize variability. Each image was displayed as a color-scale overlay on a grayscale photograph of the washers within the well plate, similar to the planktonic MRSA BLI quantification described in Sec. 2.1. The washer annulus ROI was manually constructed and used over each washer to measure BLI.

OCT imaging was performed using a spectral-domain OCT system (Ganymede II, Thorlabs GmbH, Newton, New Jersey, United States), operating at a central wavelength of 930 nm with a spectral bandwidth of 100 nm, 36 kHz scan rate, and axial resolution of 6  μm (in air). Each well plate was positioned under a table-top OCT G9 imaging probe (Thorlabs) on a 3D-printed stand, tilted 86 deg relative to the probe to avoid strong surface reflection. Imaging was conducted using the LSM03-BB objective lens (Thorlabs) with the lateral resolution of 8  μm, ∼1.2  mW of the optical power incident on the sample, focal length of 36 mm, and a transverse scan range suitable for surface imaging of biofilms. Microphotographs of each washer [Fig. 2(b)] were taken prior to imaging with a white light camera built into the OCT probe. Three-dimensional images of grown biofilm were obtained at six randomly chosen locations around each washer [Fig. 2(c)] $1.5\times 1.5\;{\mathrm{mm}}^{2}$ fields of view, containing 500×1500×1500  voxels). Images were analyzed using ThorImage software (Thorlabs). Biofilm thickness (the vertical extent of growth) was determined as the average number of pixels from the metal surface to the upper biofilm boundary over the entire volume and converted to micrometers [Fig. 2(d)] after optical path length correction using a refractive index of 1.4, commonly assumed for hydrated biofilms. OCT image scale bars were generated using the same refractive index correction to ensure they represent physical (not optical) dimensions. With biofilm thicknesses up to ∼100  μm and a $1.5\times 1.5\;{\mathrm{mm}}^{2}$ field of view, the entire imaging volume at 86 deg imaging angle remained within the 0.28 mm depth of focus of the objective lens. As such, no correction factor was applied or required for angle-related distortion. From 1500 cross-sections of each 3D OCT image, 50 were manually analyzed (every 30 microns in the lateral direction). Biofilm average thickness was approximated by taking six measurements perpendicular to the washer surface, producing 300 measurements in each area. The procedure was repeated for all six evenly spaced OCT images of the washer surface, averaged for a measure of mean thickness and multiplied by the surface area of the washer for an estimate of total biofilm volume.

### 2.3 Biofilm CFU Measurement and Density Estimation

Immediately after imaging, biofilms were prepared for CFU counting by thoroughly scraping the upward-facing surface with a surgical blade. Biomaterials were transferred into 1 mL of PBS in a 1.5 mL microcentrifuge tube (Eppendorf SE, Hamburg, Germany) and secured with Parafilm (Bemis, Inc., Sheboygan Falls, Wisconsin, United States) before vortexing for 60 s and sonication at 40 kHz for 30 min in an ultrasonic water bath with ice to release individual bacteria from the biofilm. Water bath temperature was maintained at the level of 15°C to 20°C. After sonication, the tube was vortexed for another 60 s. Multiple, 10-fold dilutions were prepared from the resulting PBS-bacteria solution and plated onto tryptic soy agar plates with 10  μL/mL chloramphenicol. Forming colonies were counted after 24 h of incubation at 37°C and expressed as total CFU per washer.

The total number of CFU (determined by plating) was divided by the biofilm volume to approximate the CFU density ($\mathrm{CFU}/{\mathrm{mm}}^{3}$) and plotted against the BLI of this biofilm.

A single drop-sized sample was taken from one of the serial dilutions of three washers chosen at random to be viewed under an inverted light microscope at 20× magnification. The number of bacterial cells that were adhered together was counted to determine the correction factor to account for any incomplete dissolution of the biofilm. Washers were cultured under standard conditions and imaged using scanning electron microscopy (SEM) to verify that these methods lead to the presence of MRSA colonies without contamination [Fig. 2(e)]. Washers imaged with SEM were not included in the standard curve data collection.

### 2.4 Bacterial Load Modelling from BLI and Density Data

The potential of BLI and sample density as predictors of CFU was evaluated by fitting log-transformed linear models to planktonic and biofilm datasets, using MATLAB’s Statistics and Machine Learning Toolbox (MathWorks, Natick, Massachusetts, United States).

The general workflow consisted of positing a linear relationship in log-space

$$\log(\mathrm{CFU})=\beta_{0}+\beta_{1}\cdot \log(\mathrm{BLI}),$$ (1)

where $\beta_{0}$ and $\beta_{1}$ are intercept and slope, respectively, representing the base amount of CFU needed to produce one BLI unit and the step-wise increase in BLI with more bacteria present. Least-squares optimization of the log–linear relationship estimated fitting parameters $\beta_{i}$ and their significance values, confidence and prediction intervals, and the model’s coefficient of determination $R^{2}$. The log–linear equation was then mathematically transformed to represent a true power–law relationship of the variables, allowing physical interpretation outside of logarithmic space

$$\mathrm{CFU}=10^{\beta_{0}}\cdot {\mathrm{BLI}}^{\beta_{1}}.$$ (2)

The performance of the proposed CFU-prediction models was assessed based on $R^{2}$, parameter significance, and the ratio between low and high residuals—distances between CFU predicted by the model versus ground truth.

## 3 Results

### 3.1 Standard Curve for Planktonic MRSA: Bioluminescence-CFU Correlation

**Fig. 3:**
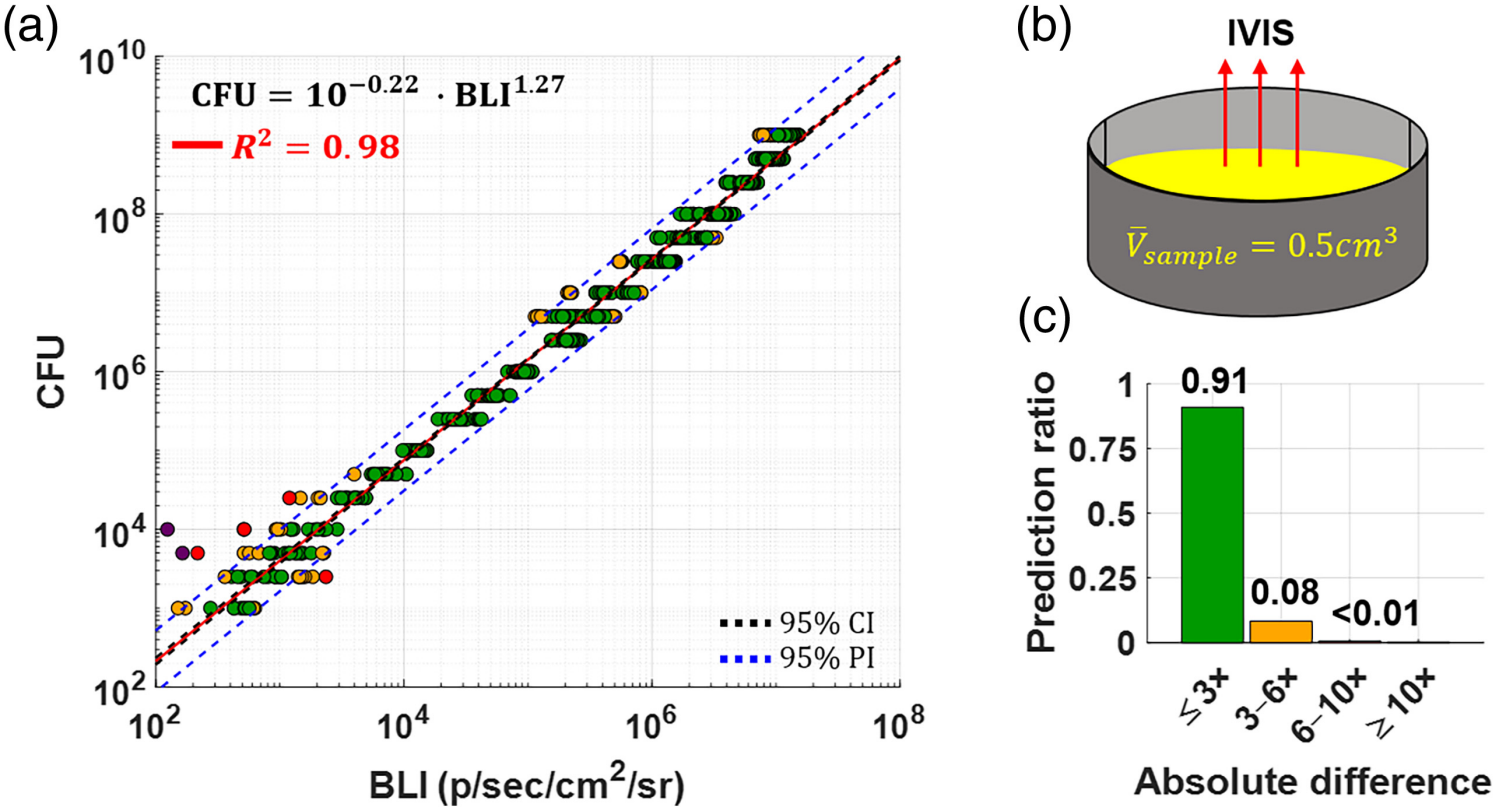
(a) Bioluminescence standard curve (red) for serial dilutions of MRSA culture of known number of CFUs in TSB; (b) schematic representation of one of the wells with planktonic NRSA from which BLI was measured, with indicated volume. Well depth was 0.7 cm, surface area was $1.77\;{\mathrm{cm}}^{2}$; (c) histogram categorizing prediction quality, summarizing ratio of accurate (green) versus highly inaccurate (orange) predictions corresponding to the data residuals.

To establish a baseline predictive model, we measured BLI from serial dilutions of planktonic MRSA SAP231-luxCDABE cultures and correlated this with CFU counts. A robust log–linear relationship was observed [Fig. 3(a)], with a coefficient of determination $R^{2}=0.98$, confirming the monotonic scaling of BLI with bacterial load across 6 orders of magnitude. The resulting model, expressed in power–law form, is displayed on the graph. Prediction intervals (blue dashed lines) span ∼1 order of magnitude, corresponding to a mean absolute error of ±66.5% in CFU estimation.

BLI was measured from cultures suspended in broth contained in 3D-printed wells with fixed volume, area, and depth [Fig. 3(b)]. Prediction quality is further summarized in Fig. 3(c) via a residual-based histogram, categorizing the accuracy of predictions. The majority of data points (green bar) fell within a factor of 2 of the predicted mean, whereas a minority showed larger deviations, with some (purple) lying more than 1 order of magnitude from the model’s estimate.

### 3.2 Poor Predictive Performance of BLI Alone in MRSA Biofilm Quantification

**Fig. 4:**
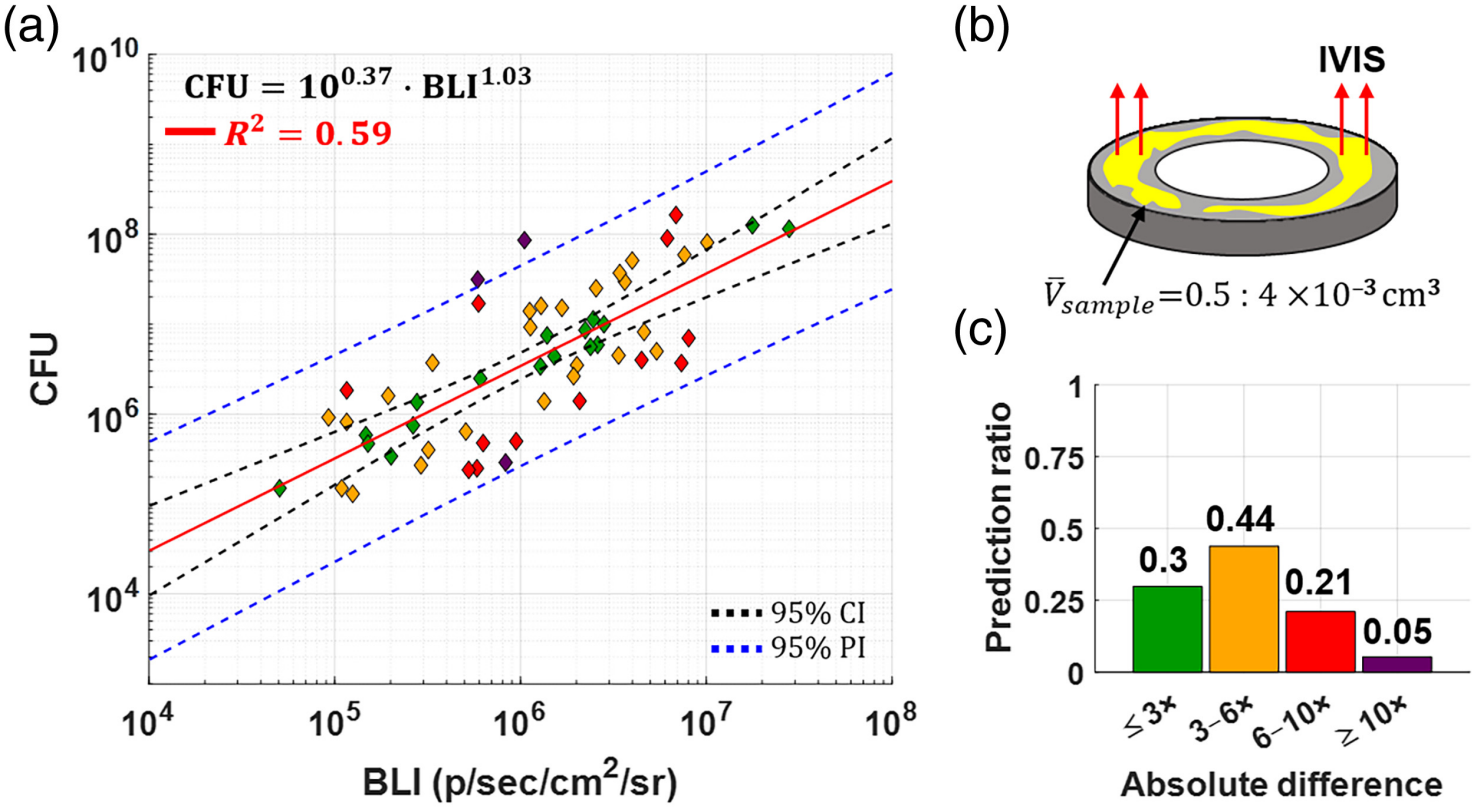
Modelling CFU of MRSA biofilm using bioluminescence: (a) Biofilm BLI versus CFU experimental data, with prediction model equation at the bottom right, showing very wide intervals for each prediction (for details see text). (b) Schematic of a metal washer with a small average biofilm volume ($0.002\;{\mathrm{cm}}^{3}$) compared with planktonic suspensions. The surface area of the metal washer was $0.94\;{\mathrm{cm}}^{2}$; (c) model performance bar plot indicates the spread of obtained biofilm data reduces the reliability of this model—most predictions made by it differ from actual, obtained CFU values by a factor of 3 or more.

We next applied the same BLI-based modeling approach to MRSA biofilms grown on stainless steel washers under flow. Although a moderate positive correlation between BLI and CFU was observed [$R^{2}=0.59$, Fig. 4(a)], the prediction intervals were extremely wide, spanning several orders of magnitude. This variability likely reflects the heterogeneous growth, structural variability, and spatial complexity of biofilms.

As illustrated in Fig. 4(b), biofilms formed compact layers of variable thickness and density on washers with an average volume of $2\;\text{m}m^{3}$. Despite controlling for inoculum, flow conditions, and time, substantial differences in structure and BLI output persisted across samples. The histogram in Fig. 4(c) shows that most predictions based solely on BLI deviated significantly from true CFU counts, with over 60% of samples falling outside the ±2-fold prediction threshold. These findings highlight the limitations of using BLI alone to quantify bacterial burden in complex 3D biofilm structures.

### 3.3 Incorporating Structural Information: OCT-Derived Volume and Bacterial Density

**Fig. 5:**
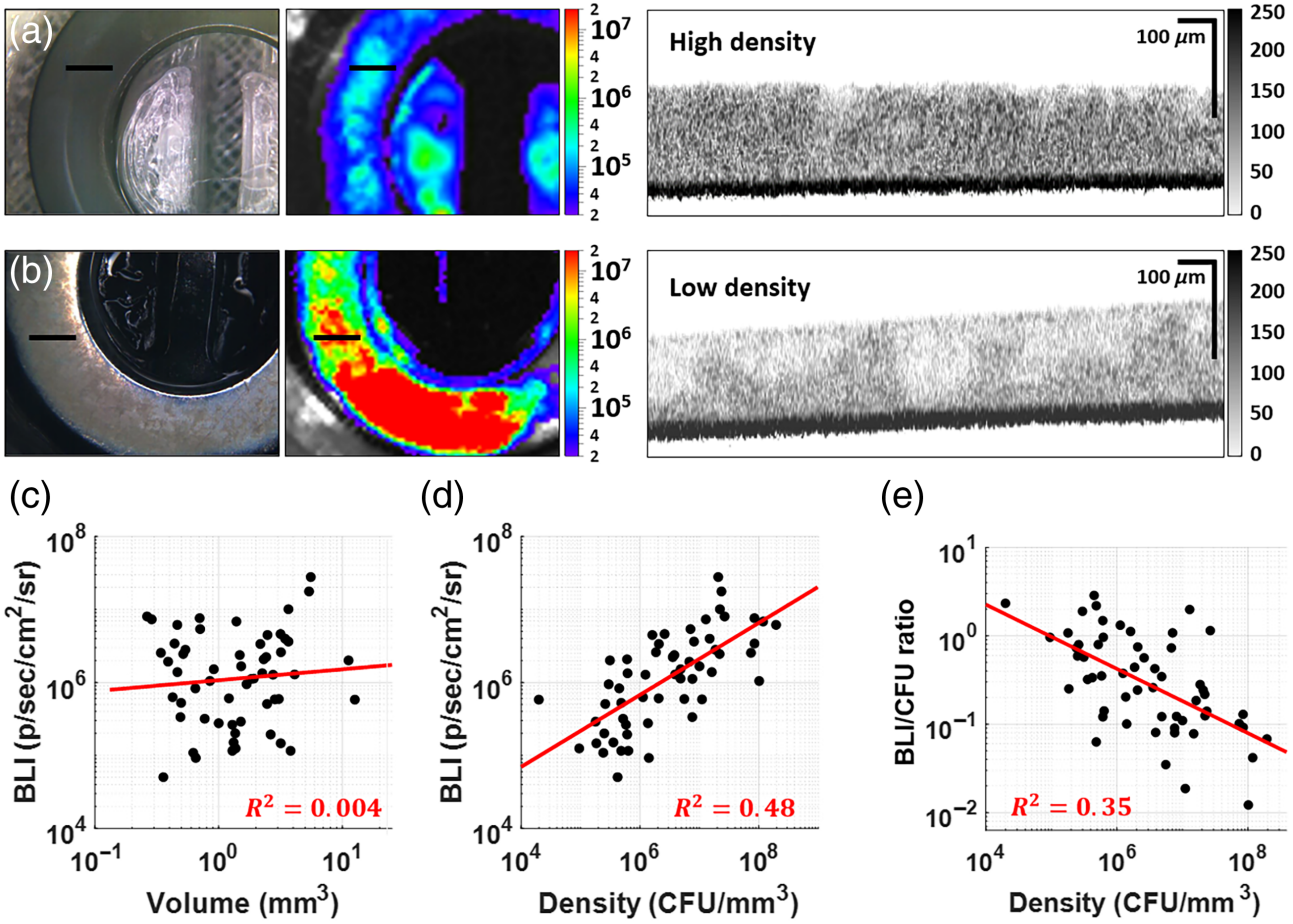
White light, bioluminescence, and cross-sectional OCT images of (a) high and (b) low density biofilms of comparable volumes but different densities and subsequent effects on produced bioluminescence signal; (c) sample volume versus BLI produced; (d) biofilm density versus its BLI; (e) biofilm density versus BLI per CFU plot.

To improve CFU prediction, we acquired OCT images of biofilm samples to assess their biovolume and structural characteristics. Figures 5(a) and 5(b) show representative white-light images, IVIS bioluminescence images, and OCT cross-sections of biofilms with similar volumes but markedly different BLI signals, suggesting that bacterial density (CFU per unit volume) may influence signal intensity. BLI alone showed a weak correlation with both biofilm volume [Fig. 5(c)] and CFU count [Fig. 4(a)] independently. However, when we related these metrics by calculating bacterial density (total CFU per imaged volume), we observed a significantly improved correlation with BLI [Fig. 5(d)]. These results indicate that the total BLI signal produced by a biofilm is influenced not only by the total number of CFUs but also by their spatial concentration within the biofilm. When comparing bacterial density to BLI output per CFU, we found a weak negative correlation [Fig. 5(e)], suggesting that in denser biofilms, metabolic downregulation may occur. This finding highlights the potential for underestimating bacterial load if BLI is interpreted without accounting for CFU density.

### 3.4 Improved CFU Prediction Models Using BLI and Bacterial Density

**Fig. 6:**
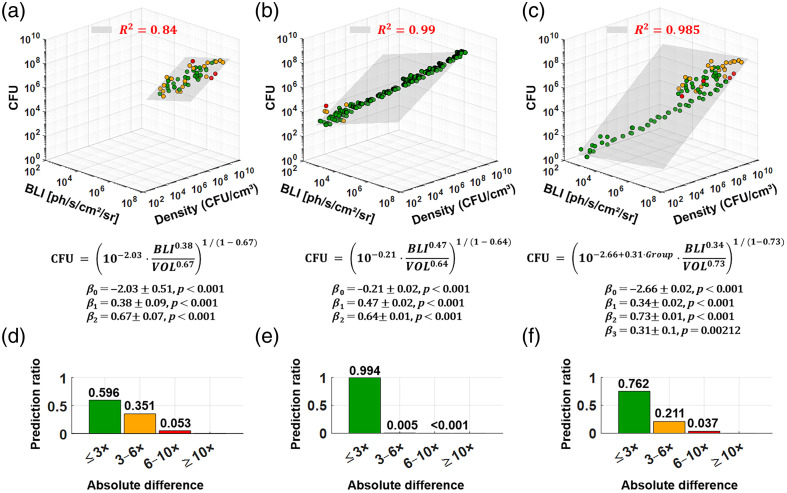
Improved CFU prediction models, using BLI and volume: (a) Biofilm predictor model, improved from $R^{2}=0.59$ to 0.84 by adding sample density. Model (gray plane of fit) is described by equation below graph; (b) similar model adjustment for planktonic bacteria; (c) joint model predicting CFU of both biofilm and planktonic MRSA; (d) prediction accuracy for panel (a) showing the proportion of points falling within defined absolute distances from the model-predicted mean CFU; (e) corresponding evaluation of model performance in panel (b) for planktonic MRSA; (f) accuracy of the joint biofilm–planktonic model. To account for differing sample sizes ($N_{\text{biofilm}}=57$, $N_{\text{planktonic}}=856$), a subset of N=57 planktonic samples was randomly selected to match the biofilm group for prediction ratio assessment.

Building on these observations, we developed a multiple linear regression model that incorporates both BLI and bacterial density to predict CFU counts. This model substantially outperformed predictions based on BLI alone, increasing the $R^{2}$ from 0.59 to 0.84 for biofilms [Fig. 6(a)]. Although the model components are algebraically related because density (DEN) is derived from CFU and volume, we reformulated the equation to predict CFU as a function of independently measured BLI and volume (VOL), thereby avoiding circular reasoning. Specifically, the identity $\log(\mathrm{CFU})=\beta_{0}+\beta_{1}\cdot \log(\mathrm{BLI})+\beta_{2}\cdot \log(\mathrm{DEN})$ was algebraically transformed using the substitution log(DEN) = log(CFU) – log(VOL), resulting in

$$\log(\mathrm{CFU})=\frac{\beta_{0}+\beta_{1}\cdot \log(\mathrm{BLI})-\beta_{2}\cdot \log(\mathrm{VOL})}{1-\beta_{2}}.$$ (3)

By rearranging and applying logarithmic and exponential rules, we derived a clearer power–law relationship that enables independent prediction of CFU based on separate measurements of bioluminescence (via IVIS) and sample volume (via OCT)

$$\mathrm{CFU}=[10^{\beta_{0}}\cdot \frac{{\mathrm{BLI}}^{\beta_{1}}}{{\mathrm{VOL}}^{\beta_{2}}}{]}^{\frac{1}{1-\beta_{2}}}.$$ (4)

The intercept ($\beta_{0}=-2.03\pm 0.51$) and both predictors BLI ($\beta_{1}=0.38\pm 0.09$) and VOL ($\beta_{2}=0.67\pm 0.07$) were statistically significant (p<0.001), indicating that both metabolic activity and structural biofilm parameters contribute to the estimation of bacterial load CFU.

When applied to planktonic samples, for which density was defined as CFU per milliliter of broth, the model similarly improved predictive power [$R^{2}=0.99$; Fig. 6(b)], reducing prediction errors to within a ±50% range for nearly all samples. A multiple linear regression model predicting CFU from BLI and VOL in planktonic cultures yielded $\beta_{0}=-0.21\pm 0.02$, $\beta_{1}=0.47\pm 0.02$, and $\beta_{2}=0.64\pm 0.01$ (all p<0.001), indicating a strong and consistent relationship among bioluminescence signal, culture density, and bacterial burden. The joint model using normalized BLI and density values across both biofilm and planktonic samples [Fig. 6(c)] yielded $\beta_{0}=-2.66\pm 0.02$, $\beta_{1}=0.34\pm 0.02$, $\beta_{2}=0.73\pm 0.01$, and $\beta_{3}=0.31\pm 0.10$ (all p<0.001 except $\beta_{3}$, p=0.002). Although the joint model demonstrated robust CFU prediction across heterogeneous samples with mixed structural features [Fig. 6(f)], the inclusion of a binary group variable (0 = planktonic, 1 = biofilm) revealed a statistically significant effect (p<0.05), indicating that the relationship between predictors and CFU differs significantly between planktonic and biofilm conditions. This finding underscores the need for a more advanced model that accounts for the distinct characteristics of each region to improve prediction accuracy. Such a model would require segmentation of planktonic and biofilm subvolumes based on yet-to-be-defined OCT-derived biomarkers, along with correlation of BLI + subvolumes measurements with CFU counts in mixed environments. Taken together, these results demonstrate that integrating BLI with OCT-derived structural metrics, such as biofilm volume, enables calculation of bacterial density when combined with CFU measurements, providing a significantly more accurate and generalizable framework for noninvasive quantification of bacterial load.

## 4 Discussion

This study demonstrates that integrating BLI with structural metrics derived from OCT markedly improves the accuracy of bacterial load estimation in both planktonic and biofilm-associated MRSA. Using the luxCDABE-tagged MRSA strain SAP231, we confirmed a strong, monotonic log–log relationship between BLI signal and CFU count in planktonic cultures, consistent with previous reports in various luciferase constructs, including *Pseudomonas aeruginosa*, *Escherichia coli*, and *Listeria monocytogenes*.^17^^,^^18^ The luxCDABE operon has been widely adopted in microbial pathogenesis and infection models for semi-quantitative tracking of bacterial load,^19^^,^^20^ and our findings further validate its use in MRSA. Importantly, uniform ROI positioning and exposure settings ensured high reproducibility and minimized technical variability, consistent with recommendations from prior BLI standardization efforts.^21^ The simple power–law model achieved high predictive accuracy ($R^{2}=0.98$ to 0.99) across a wide dynamic range, validating BLI as a reliable noninvasive readout of viable bacterial burden under well-mixed conditions.

However, applying the same approach to biofilms revealed significant limitations. BLI-based CFU prediction in biofilms was much less reliable ($R^{2}=0.59$), with wide confidence intervals and high residual errors largely due to the spatial and metabolic heterogeneity of biofilm structures. Even with matched inocula and flow conditions similar to macrofluidic systems that have been used to simulate clinically relevant biofilm environments on medical device analogs,^22^^,^^23^ OCT imaging revealed considerable variation in thickness, density, and layering. These differences affect both optical transmission of BLI signal^24^ and bacterial metabolic output,^25^^,^^26^ confounding simple correlation with CFU counts. In denser biofilms, we observed reduced BLI per CFU likely due to metabolic quiescence in hypoxic microenvironments, a well-documented phenomenon in mature or treatment-resistant biofilms.^27^ This underscores the importance of accounting for structural and physiological context when interpreting BLI data in biofilm research or diagnostics.

To address these challenges, we incorporated OCT-derived metrics, specifically biofilm volume and bacterial density as factors in our modeling framework. We derived a physically meaningful power–law model to independently estimate CFU without circular logic, by algebraically decomposing density into separately measurable terms. This IVIS-OCT dual-modality approach significantly improved model performance ($R^{2}=0.84$), highlighting CFU density as a key factor influencing BLI output. However, the inclusion of a binary group variable (planktonic versus biofilm) revealed a significant interaction effect (p<0.05), indicating that the relationships between predictors and CFU differ fundamentally between growth modes. These findings underscore the need for future models to account for intrinsic differences between planktonic and biofilm states, not only at the whole-sample level but also within spatially heterogeneous environments. In rodent or large animal trauma models, infection sites often contain intermixed bacterial communities in diverse metabolic states. Thus, a more advanced, spatially resolved modeling approach will be essential for accurately quantifying bacterial burden in such complex settings.

Development of such a model will require automated segmentation of biofilm versus planktonic regions within 3D OCT volumes, enabling region-specific application of tailored prediction equations. Simple OCT signal thresholding (biofilms typically exhibit higher backscatter intensity due to their dense matrix) could serve as a first-pass segmentation method in high-SNR datasets. More robust differentiation could be achieved through second-order texture metrics (e.g., entropy, contrast, correlation), which are sensitive to the structural heterogeneity characteristic of biofilms.^15^^,^^28^ These methods could capture microstructural features such as pores, layering, or roughness. In addition, analysis of speckle decorrelation rates over time may reveal biomechanical differences between static biofilm biomass and more mobile planktonic fluid. Previous work in OCT angiography^29^ and elastography^30^ suggested that speckle variance analysis can resolve dynamic tissue properties at high spatial resolution. Each of these approaches presents a feasible route toward the automated classification of subvolumes, which could then be correlated with local BLI intensity and ground-truth CFU measurements, supporting development of subregion-specific regression models. This would enable more realistic modeling of heterogeneous infection landscapes *in vivo* and ultimately inform diagnostics or therapeutic monitoring.

Because this approach leverages bioluminescence rather than fluorescence, it avoids interference from tissue or bacterial autofluorescence and does not require external excitation, making it particularly advantageous for *in vivo* imaging. Although this study focused on a lux-tagged MRSA strain, similar constructs have been developed for a wide range of pathogens, allowing this method to be extended to other bacterial species. In human applications, bioluminescent reporters are not feasible. Therefore, future translational work must assess whether alternative surrogate metrics, such as fluorescence-tagged antibodies, metabolic fluorophores, or endogenous OCT contrast features (e.g., attenuation rate, birefringence, or angiographic signal), can replicate the predictive utility of lux-based BLI. Correlation with *ex vivo* CFU quantification and clinical microbiology data will be essential for validating these approaches. Multimodal imaging strategies are actively being developed to combine label-free optical techniques such as OCT with fluorescence or bioluminescence, enabling more comprehensive biofilm characterization.^4^^,^^16^^,^^31^ Recently, we and others have demonstrated the application of OCT for nondestructive imaging of mono- and mixed-species biofilms in clinical contexts, underscoring its promise for translational use.^32^^,^^33^

Although the present BLI–OCT platform is not directly applicable to human use due to the requirement for genetically encoded luminescent strains, it provides an effective tool for preclinical evaluation of antimicrobial therapies in animal models. This method was developed in response to challenges encountered during *in vivo* studies of photodynamic therapy (PDT) in rat and rabbit orthopedic infection models, where BLI alone proved insufficient for accurately tracking bacterial burden in dense or metabolically inactive biofilms. By integrating metabolic and structural data, the system enables noninvasive, longitudinal assessment of treatment response and biofilm dynamics in preclinical settings, supporting the development and refinement of anti-biofilm strategies.

One limitation of this approach is the reliance on metabolic activity for photon production, as lux operon expression is ATP- and redox-dependent. Consequently, BLI may underestimate viable but metabolically inactive populations, particularly in hypoxic or nutrient-depleted zones, a limitation shared with other viability-dependent readouts such as resazurin reduction assays.^34^^,^^35^ Moreover, although IVIS provides excellent whole-sample visualization, it lacks the spatial resolution to discern microscale heterogeneity within the biofilm. The observed spatial variations in BLI intensity across the washer likely reflect local differences in bacterial metabolic activity and viability driven by microenvironmental gradients such as nutrient and oxygen availability. By contrast, OCT measures average biofilm thickness, providing a structural metric that assumes uniform coverage over the washer surface. This assumption is a simplification, as biofilm thickness can vary locally. Nonetheless, total CFU enumeration integrates bacterial viability over the entire biofilm volume, capturing overall population size despite such heterogeneity. Future work may incorporate high-resolution bioluminescence microscopy or spatial transcriptomics to map metabolic gradients and lux activity at the cellular level.^36^

Overall, our findings support the use of bioluminescent MRSA strains and IVIS-based BLI as reliable, semi-quantitative tools for monitoring bacterial burden in both planktonic and biofilm-associated states. When coupled with structural imaging modalities such as OCT, this platform offers a powerful, noninvasive method for evaluating biofilm responses to antimicrobial interventions.

## 5 Conclusion

This study demonstrates the feasibility and value of combining bioluminescence imaging with OCT to quantitatively assess MRSA in both planktonic and biofilm forms. By integrating functional and structural optical readouts, our IVIS-OCT platform enables noninvasive scalable evaluation of bacterial viability and biofilm architecture. The use of a macrofluidic MRSA biofilm model faithfully mimics clinically relevant features of hardware-associated infections and provides a robust tool for preclinical therapeutic testing. These findings directly support the advancement of photodynamic therapy and other optical antimicrobial strategies, underscoring the translational power of integrated biophotonics approaches.

## Data Availability

The datasets generated and analyzed during the current study are not publicly available but may be obtained from the corresponding author upon reasonable request.
